# Schwann Cell Expressed Nogo-B Modulates Axonal Branching of Adult Sensory Neurons Through the Nogo-B Receptor NgBR

**DOI:** 10.3389/fncel.2015.00454

**Published:** 2015-11-23

**Authors:** Christoph Eckharter, Nina Junker, Lilli Winter, Irmgard Fischer, Barbara Fogli, Steffen Kistner, Kristian Pfaller, Binhai Zheng, Gerhard Wiche, Lars Klimaschewski, Rüdiger Schweigreiter

**Affiliations:** ^1^Division of Neurobiochemistry, Biocenter, Innsbruck Medical UniversityInnsbruck, Austria; ^2^Max F. Perutz Laboratories, Department of Biochemistry and Cell Biology, University of ViennaVienna, Austria; ^3^Department of Anatomy, Histology and Embryology, Division of Neuroanatomy, Innsbruck Medical UniversityInnsbruck, Austria; ^4^Department of Anatomy, Histology and Embryology, Division of Histology and Embryology, Innsbruck Medical UniversityInnsbruck, Austria; ^5^Department of Neurosciences and Biomedical Sciences Graduate Program, School of Medicine, University of California, San DiegoLa Jolla, CA, USA

**Keywords:** nogo, schwann cells, sensory neurons, morphology, axonal branching, receptor, sciatic nerve, knockout

## Abstract

In contrast to the central nervous system (CNS) nerve fibers do regenerate in the peripheral nervous system (PNS) although in a clinically unsatisfying manner. A major problem is excessive sprouting of regenerating axons which results in aberrant reinnervation of target tissue and impaired functional recovery. In the CNS, the reticulon protein Nogo-A has been identified as a prominent oligodendrocyte expressed inhibitor of long-distance growth of regenerating axons. We show here that the related isoform Nogo-B is abundantly expressed in Schwann cells in the PNS. Other than Nogo-A in oligodendrocytes, Nogo-B does not localize to the myelin sheath but is detected in the ER and the plasma membrane of Schwann cells. Adult sensory neurons that are cultured on *nogo-a/b* deficient Schwann cells form significantly fewer axonal branches vs. those on wildtype Schwann cells, while their maximal axonal extension is unaffected. We demonstrate that this effect of Nogo-B on neuronal morphology is restricted to undifferentiated Schwann cells and is mediated by direct physical contact between these two cell types. Moreover, we show that blocking the Nogo-B specific receptor NgBR, which we find expressed on sensory neurons and to interact with Schwann cell expressed Nogo-B, produces the same branching phenotype as observed after deletion of Nogo-B. These data provide evidence for a novel function of the *nogo* gene that is implemented by the Nogo-B isoform. The remarkably specific effects of Nogo-B/NgBR on axonal branching, while leaving axonal extension unaffected, are of potential clinical relevance in the context of excessive axonal sprouting after peripheral nerve injury.

**Main Points:**

- Nogo-B is prominently expressed in Schwann cells and localizes to the ER and plasma membrane. It distributes to the external cytoplasmic compartment of Schwann cells *in vivo*, but is absent from the myelin sheath.
- Genetic deletion of Nogo-B in Schwann cells reduces axonal branching, but not long-distance growth, of co-cultured adult sensory neurons.
- Schwann cell expressed Nogo-B interacts with neuronal NgBR. Blockade of NgBR mimics the loss-of-*nogo* branching phenotype.

## Introduction

Injury to peripheral nerves elicits a series of cellular and molecular transformations distal to the site of injury called Wallerian degeneration, which paves the way for regenerative outgrowth of lesioned nerve fibers (Chen et al., 2007; Faroni et al., 2015). Key players in Wallerian degeneration are Schwann cells, which are the main glial cell type in the peripheral nervous system (PNS; Navarro et al., 2007). Within hours after receiving a nerve lesion they start to dedifferentiate by detaching from the axons and retracting the myelin sheath. They phagocytose axonal and myelin debris and chemotactically recruit blood-borne macrophages for assistance. During this phase of clearance and resorption, Schwann cells enter the cell cycle and gradually form what has become known as bands of Büngner, an endoneurial cellular scaffold that provides essential guidance and support for regenerating axons. While the cocktail of neurotrophic factors and cytokines that is secreted by Schwann cells in this phase of repair promotes the survival of neurons and axonal elongation, it concomitantly favors axonal sprouting (Klimaschewski et al., 2013). Each transected axon can sprout up to 25 branches from the proximal stump, which ultimately innervate multiple target sites rather than the single original site (Mackinnon et al., 1991). This aberrant reinnervation affects motor, sensory and sympathetic fibers alike and severely compromises functional recovery due to asynchronized motor function and sensory deficits (Hendry et al., 1986; Sumner, 1990; de Ruiter et al., 2008; Allodi et al., 2012). Enhancing axonal elongation by exogenous application of neurotrophic factors or through electrical stimulation at the same time increases the extent of axonal sprouting and aberrant reinnervation of peripheral targets (English, 2005; Klimaschewski et al., 2013). From a clinical point of view it is thus highly desirable to identify mechanisms that distinguish between long-distance growth and sprouting of regenerating nerve fibers. One approach to specifically promote axonal elongation without increasing axonal sprouting is to disentangle the signaling activities triggered by neurotrophic factors and cytokines and to modulate individual signaling cascades such that elongation is stimulated but sprouting remains unaffected (Hausott et al., 2009; Marvaldi et al., 2015). Here, we report a completely novel mechanism to distinguish between axonal elongation and branching that is mediated by Schwann cell expressed Nogo-B and its neuronally expressed receptor NgBR. In a co-culture of Schwann cells and adult sensory neurons, we demonstrate that blocking the Nogo-B/NgBR signaling axis reduces the number of axonal branches but does not compromise axonal elongation.

## Materials and Methods

### Animals

The *nogo-a/b* deficient mouse line lacked the isoforms Nogo-A and -B due to targeting of the first exon containing the start codon. This mouse line was described elsewhere (Zheng et al., 2003). Littermates were used for experiments that involved different *nogo* genotypes. In addition, Schwann cells and sensory neurons were obtained from C57Bl/6N mice. A pair of rat sciatic nerves was obtained from one young adult Sprague-Dawley rat. All experimental protocols were approved by the Austrian Animal Experimentation Ethics Board in compliance with the European Convention for the Protection of Vertebrate Animals Used for Experimental and other Scientific Purposes (ETS no. 123).

### Immunohistochemistry

Mice were anesthetized with isoflurane and perfused with 4% PFA/PBS. Sciatic nerves were dissected and immersion fixed in 4% PFA/PBS for 2 h at RT. Tissues were mounted in OCT compound, snap-frozen in isopentane cooled with liquid nitrogen, and thin sections (3 μm) were obtained on a Microm Microtom Cryostat Hm500OM. Sections were blocked with 2% BSA/PBS for 30 min at RT and incubated with the following primary antibodies diluted in PBS/0.1% Tween-100 for 1 h at RT: rabbit Bianca antiserum (1:2000; Oertle et al., 2003b), mouse myelin basic protein (MBP; 1:200; Chemicon #MAB386), chicken neurofilament antibody (NF-H; 1:2000; Acris #CH22104). Sections were rinsed thoroughly in PBS and then incubated with secondary antibodies (Alexa Fluor 488 donkey anti-rabbit, Alexa Fluor 568 goat anti-chicken, Thermo Fisher, 1:500; Cy5 donkey anti-rat, Jackson ImmunoResearch, 1:500) for 1 h at RT. After washing with PBS, nuclei were stained with Hoechst (1:3000) for 5 min at RT. Slides were washed in PBS, water, and air-dried before mounting with Mowiol. Microscopy was performed using a LSM710 confocal laser scanning microscope (Zeiss) equipped with a Plan-Apochromat 63× 1.4 NA objective lens. Images were obtained using the LSM710 module and the Zeiss ZEN software.

**Figure 1:**
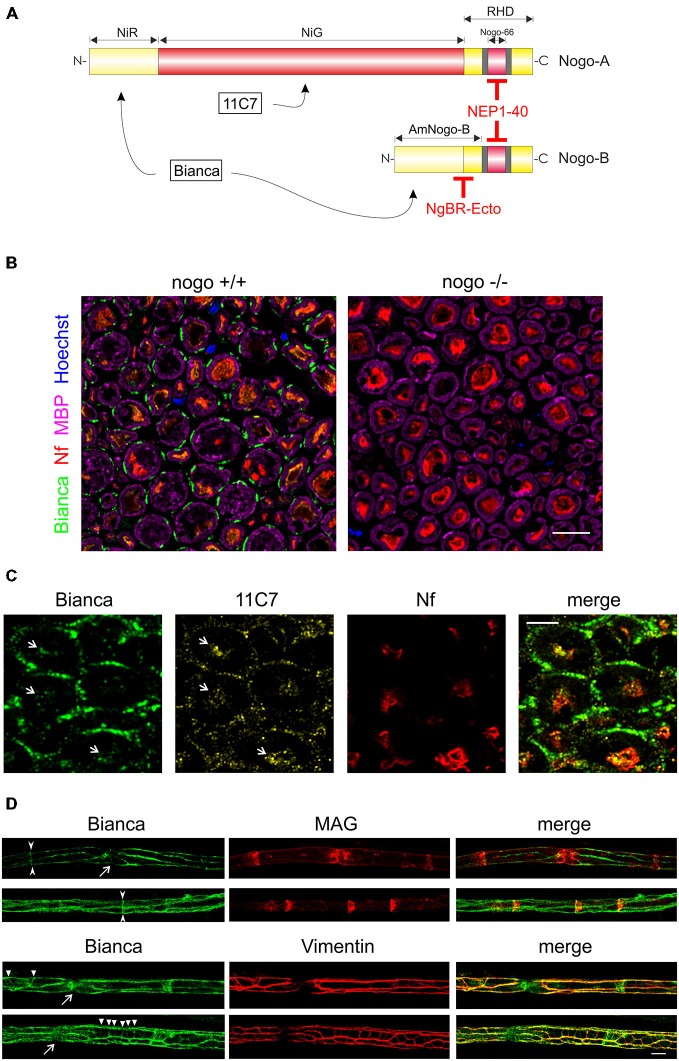
**Immunohistochemistry of Nogo-B in the sciatic nerve. (A)** The two isoforms Nogo-A and Nogo-B differ considerably in size and function. A common feature is the C-terminal RHD which harbors not only an ER anchoring motif at the very C-terminus but also two hydrophobic sequence stretches (shaded in gray); they are believed to contribute to ER tubulation by inducing membrane curvature. Nogo-A is selectively recognized by the monoclonal 11C7 antibody while the epitope of the Bianca antiserum resides within the NIR domain that is shared by both isoforms. The NEP1–40 peptide is derived from and blocks the signaling activities of the Nogo-66 loop domain. The Nogo-B specific receptor NgBR, and recombinant NgBR-Ecto, bind to the AmNogo-B sequence stretch that is composed of parts of the NIR domain and RHD. **(B)** Immunolabeling of cross sections of the sciatic nerve of a wildtype and *nogo-a/b* knockout mouse. The external cytoplasmic compartment of Schwann cells is immunoreactive for Nogo. The myelin sheath that is visualized with myelin basic protein (MBP) is devoid of Nogo. Axons are revealed with a neurofilament antibody. Single confocal sections are shown. Scale bar is 10 μm. **(C)** Immunolabeling of cross sections of the rat sciatic nerve with the Bianca antiserum and counter-staining with 11C7. Schwann cells are strongly positive for Bianca but not for 11C7 indicating that the majority of Bianca immunoreactivity is due to Nogo-B. In contrast, the Bianca immunoreactivity in axons seems to be due to Nogo-A as demonstrated by the 11C7 signal (white arrows). Scale bar is 5 μm. A single confocal section is shown. **(D)** Immunolabeling of teased nerve fibers of the sciatic nerve of an adult wildtype mouse. There is prominent Nogo immunoreactivity in Cajal bands and, to a lesser extent, in Schmidt-Lanterman incisures (arrow heads) and transverse trabeculae (triangles) as revealed by MAG and Vimentin co-distribution. The Nogo staining typically extends into nodes of Ranvier (arrows). Single confocal sections are shown. Scale bar is 10 μm.

For the differential Bianca/11C7 staining (for epitopes see Figure 1A) we prepared sciatic nerve from adult rat because of unspecific signals obtained with the 11C7 antibody with mouse tissue. Sciatic nerves were fixed overnight at 4°C in a PBS based solution of 2% PFA and 15% picric acid (Zamboni’s fixative), cryoprotected in 20% sucrose for 72 h at 4°C, embedded in Tissue-Tek O.C.T (Sakura #4583) and rapidly frozen in freezing isopentane. Nerve cross-sections of 8 μm thickness were permeabilized with 0.3% Triton X-100 and blocked with 2.5% horse serum in PBS for 1 h at RT. Sections were incubated o/n at 4°C with the following primary antibodies: chicken neurofilament antibody (NF-H; 1:3000; Acris #CH22104), rabbit Bianca antiserum (1:10,000), mouse Nogo-A 11C7 antibody (1:5000; Oertle et al., 2003b), all diluted in blocking buffer. Sections were rinsed three times in PBS and incubated with secondary antibodies (Alexa Fluor 488 goat anti-mouse, Alexa Fluor 568 goat anti-chicken, Alexa Fluor 647 goat anti-rabbit; Thermo Fisher; 1:1000) for 2 h at room temperature. Nuclei were stained with Hoechst (1:20,000 in water) for 30 min at RT before slides were rinsed and embedded with aqueous mounting medium (Dako #S3023). Images were acquired with a HCX PL APO lambda blue 63.0×, 1.40 NA, oil immersion objective on a Leica TCS SP5 laser scanning confocal microscope.

Preparation and immunostaining of teased sciatic nerve fibers was essentially performed as described in Walko et al. (2013). Mice were anesthetized with isoflurane and then perfused with 4% PFA/PBS. Sciatic nerves were prepared and immersion fixed for 20 min at RT. After washing with PBS, nerves were teased on Superfrost plus glass slides and air-dried. The staining procedure was performed using the M.O.M. Basic Kit (Vector Laboratories #BMK-2202) according to the manufacturer’s instructions using the following primary and secondary antibodies: rabbit Bianca antiserum (1:2000), mouse MAG antibody (1:100; Chemicon #MAB1567), goat Vimentin antiserum (1:400; Giese and Traub, 1986), DyLight550 donkey anti-rabbit and Alexa Fluor 488 donkey anti-goat (1:500; Thermo Scientific); for primary mouse monoclonal antibodies the biotinylated secondary antibodies from the M.O.M. Kit in combination with Strepatividin RRX (Jackson ImmunoResearch #016-290-084) were used. All dilutions were performed in 0.1% Triton/PBS. Microscopy was performed using a LSM710 confocal laser scanning microscope (Zeiss) equipped with a Plan-Apochromat 63× 1.4 NA objective lens. Images were obtained using the LSM710 module and the Zeiss ZEN software.

### Schwann Cell and Sensory Neuron Co-Culture

Schwann cell cultures were prepared from p3–4 mice pups according to (Wrabetz et al., 1995) with minor modifications. Sciatic nerves were dissected and incubated at 37°C in 5 mg/ml collagenase type I (Thermo Fisher #17100-017) for 7 min and in 0.25% trypsin (Sigma #T4549) for 5 min. Trypsin was neutralized with DMEM high glucose/10% fetal bovine serum/1×glutamine/1× penicillin/streptomycin. Two rounds of trituration were carried out with a flame-polished Pasteur pipette in the presence of 10 μg/ml DNase I (Roche #11284932001). Pooled cell suspensions were centrifuged at 290× g for 10 min and the cell pellet was resuspended in DMEM high glucose/10% fetal bovine serum/1×glutamine/1×penicillin/streptomycin. Schwann cells were plated onto glass cover slips coated with poly-_L_-lysine (Sigma #P4707) at a density of 1.25 × 10^5^ cells cm^−2^ and incubated o/n at 8.5% CO_2_. The following day (DIV1), medium was changed to serum-free PNGM-A medium (Bullet Kit; Lonza #C4512) plus 2 μM cytosine arabinoside (CA; Sigma #C1768) and dishes were placed at 5% CO_2_.

Sensory neurons were obtained from thoraco-lumbal dorsal root ganglia (DRG) of either adult mice of the age of 2–3 months or of early postnatal p6–9 pups. Dissection and dissociation of ganglia were previously described in Schweigreiter et al. (2004). Briefly, ganglia were incubated twice at 37°C in 0.25 mg/ml Liberase DL (Roche #05401160001) for 35 min and once in 0.05% trypsin for 30 min. Ganglia were triturated in two rounds with DMEM high glucose using a flame-polished Pasteur pipette. The pooled cell suspension was centrifuged through a 3.5% BSA cushion (Sigma #A7906) in a swing-out rotor at 14× g for 15 min. The cell pellet was resuspended in PNGM-A medium and neurons were added to the Schwann cell culture at a low density of 1200 cm^−2^. After 13.5–14 h, at DIV2, the co-culture was fixed for immunocytochemistry.

For the indirect co-culture experiments Schwann cells were plated onto the bottom of a cell culture dish. Sensory neurons were plated in PNGM-A at a density of 2000 cm^−2^ onto glass cover slips that were coated with poly-_L_-lysine and laminin (Sigma #L2020) and that had tiny paraffin (Sigma #6330) feet attached. Neurons were allowed to adhere for 1.5 h before transferring the cover slips neuron-side-down onto the Schwann cell culture. The distance between the Schwann cell monolayer and the cover slip-attached neurons was approximately 0.8 mm.

For analyzing differentiating Schwann cells, 2 μM forskolin (Sigma #F3917) was added to the Schwann cell culture at DIV1 and sensory neurons were added at DIV4. The co-culture was fixed at DIV5.

For blocking experiments C57Bl/6N mice were used for both Schwann cells and sensory neurons. Blocking agents, NEP1–40 (Sigma #N7161) at 1 μM final concentration or NgBR-Ecto (Cusabio #CSB-MP860384MO) at 1 μg/ml final concentration were added to the Schwann cell culture at DIV1 shortly before neurons.

### Plasma Membrane Preparation

Plasma membrane sheets of cultured Schwann cells were enriched according to (Olpe et al., 1990). Schwann cells were harvested at DIV2. Briefly, cells were washed with PBS and dounced in homogenization buffer (0.32 M sucrose, 1 mM MgCl_2_, 1 mM K_2_HPO_4_, plus protease inhibitors). The homogenate was centrifuged in a swing-out rotor with 760× g for 5 min at 4°C. The pellet was homogenized and centrifuged for a second time. The pooled supernatants represented the postnuclear supernatant (PNS). The PNS was centrifuged with 18,000× g for 15 min at 4°C resulting in a pellet and the microsomal supernatant (MS). The pellet was resuspended and osmotically shocked in 350 μl icecold water and, following an incubation period on ice for 30 min, centrifuged with 39,000× g for 30 min at 4°C yielding a pellet of membrane sheets and the first membrane washing supernatant (MWS1). The pellet was resuspended in 350 μl Krebs-Henseleit buffer (KHB; 118 mM NaCl, 4.7 mM KCl, 1.2 mM MgSO_4_, 1.3 mM CaCl_2_, 11 mM glucose, 1.2 mM KH_2_PO_4_, 25 mM NaHCO_3_, 10 mM Hepes pH 7.35) and kept at −20°C for 2 days. After thawing at RT and centrifuging with 18,000× g for 15 min at 4°C a pellet and the second membrane washing supernatant (MWS2) were obtained. The pellet was subjected to three more washing steps with KHB as described. After the third washing step the pellet was resuspended in KHB and left o/n at 4°C. The membrane sheet pellet was washed again 3× with KHB and finally the plasma membrane enriched fraction (PM) was dissolved in 100 μl of 1× Laemmli buffer.

### Surface Biotinylation

Surface biotinylation of total protein was performed according to (Mammen et al., 1997) with the following modifications. Schwann cells at DIV2 were washed with PBS before 1 mg/ml of Sulfo-NHS-SS-Biotin (Thermo Fisher #21338) was added. After incubating at RT for 12 min the crosslinker was quenched for 3 × 5 min with PBS/0.5% BSA/50 mM glycine pH 7.4. Cells were washed with phosphate buffer (10 mM NaPO_4_, 5 mM EDTA, 5 mM EGTA, 100 mM NaCl plus protease and phosphatase inhibitors, pH 7.4) and lysed in phosphate buffer plus 0.17% SDS/0.83% Triton X-100. Following sonification for 30 s at medium intensity the cell lysate was pelleted for 5 min at 20,000× g. The supernatant (input sample) was incubated with pre-blocked NeutrAvidin sepharose (Thermo Fisher #53150) for 2 h at 4°C. The post-incubation supernatant represented the flowthrough sample. The sepharose beads were washed 3 × with phosphate buffer plus 1% Triton X-100, 2 × with phosphate buffer plus 1% Triton X-100/500 mM NaCl, and 1 × with phosphate buffer only. Finally, bound proteins were eluted by boiling the beads in 1 × Laemmli buffer.

### Immunoprecipitation and Western Blotting

Schwann cell/sensory neuron co-cultures or Schwann cells only were lysed after indicated time periods as described previously in Oertle et al. (2003b). For co-immunoprecipitation experiments lysis supernatant was either used as input control or it was incubated with Bianca antiserum (Oertle et al., 2003b) or a rabbit control IgG (Sigma #I5006) for 6 h at 4°C. Protein A/G agarose (Thermo Fisher #20421) was added and incubated o/n at 4°C. After washing with lysis buffer (Oertle et al., 2003b), bound proteins were eluted by boiling in 1× Laemmli buffer. Samples were subjected to SDS-PAGE (10%) and proteins were blotted onto a PVDF membrane (GE Healthcare #RPN303F). For detection the following primary antibodies were used: rabbit Bianca antiserum (1:20,000), rabbit NgBR antibody (1:400; proteintech #15986-1-AP), rabbit PO antibody (1:500; proteintech #10572-1-AP), rabbit p75^NTR^ antibody (1:1000; Promega #G3231), mouse actin antibody (1:10,000; Chemicon #MAB1501), mouse L-caldesmon (1:500; BD Biosciences #610660). Membranes were incubated with primary antibodies o/n at 4°C in blocking solution, 3.5% skim milk in TBS-T_WB_ (10 mM Tris, 150 mM NaCl, and 0.2% Tween 20, pH 8.0). After washing with TBS-T_WB_, membranes were incubated with secondary antibodies (IRDye 680RD goat anti-mouse IgG, LI-COR #926-68070; IRDye 800CW goat anti-rabbit IgG, LI-COR #926-32211; each at 1:20,000) in blocking solution for 2 h at RT. After washing with TBS-T_WB_, membranes were scanned with an LI-COR Odyssey Infrared Imaging System. Western Blot signals were densitometrically quantified by measuring their integrated intensity using the Odyssey software.

### Scanning Electron Microscopy

Cells grown on Thermanox coverslips (Nunc #174950) were fixed with 2.5% glutardialdehyde in 0.15 M cacodylate buffer (pH 7.3) for 24 h. A brief washing in the same buffer was followed by 1 h of postfixation with 1% aqueous OsO_4_, gradual dehydration with ethanol, and critical point drying using a Bal-Tec CPD 030 from Oerlikon Balzers. Specimens were mounted with Leit C (Plano #G3302) onto aluminium stubs, sputtered with 10 nm Au/Pd, and examined with a Zeiss field emission scanning electron microscope (DSM 982 Gemini).

### Immunocytochemistry

Cells were fixed with 4% PFA/5% sucrose in PBS for 20 min at RT, washed with PBS, incubated in Blocking Solution (3% normal goat serum, 1% BSA, 0.1% Triton X-100, in PBS) for 1 h at RT before primary antibodies were added o/n at 4°C as indicated: rabbit Bianca antiserum (1:10,000), mouse 11C7 Nogo-A antibody (1:1000), rat NCAM antibody (H28; 1:100; Santa Cruz #sc-59934), chicken calreticulin antibody (1:1000; Abcam #ab14234), rabbit S100 antiserum (Dako #Z0311), mouse neurofilament antibody (N52; 1:500; Sigma #N0142). After washing with TBS-T_IF_ (10 mM Tris, 150 mM NaCl, and 0.1% Tween 20, pH 8.0) samples were incubated with secondary antibodies (Alexa Fluor 488 goat anti-mouse, Alexa Fluor 488 goat anti-rabbit, Alexa Fluor 647 goat anti-rat, Alexa Fluor 568 goat anti-chicken; 1:1000; Thermo Fisher) in Blocking Solution for 2 h at RT. Cells were washed with TBS-T_IF_ and water plus Hoechst (1:10,000), and embedded in Fluorescent Mounting Medium (Dako #S3023). For NgBR staining fixed co-cultures were blocked with 3% BSA/TBS-T_IF_ for 1 h at RT before goat NgBR antibody (1:100; Santa Cruz #sc-138044) and mouse neurofilament antibody (N52; 1:500) were added for o/n incubation at 4°C. After washing with TBS-T_IF_ samples were incubated with secondary antibodies (Alexa Fluor 568 donkey anti-goat, Alexa Fluor 488 goat anti-mouse; 1:1000; Thermo Fisher) in 3% BSA/TBS-T_IF_ for 2 h at RT, washed and embedded. For surface stainings, cells were fixed with 4% PFA/5% sucrose in PBS for 20 min at RT, washed with PBS, incubated in Blocking Solution (3% normal goat serum, 1% BSA, in PBS) for 1 h at RT before primary antibodies were added o/n at 4°C as described above. After washing with PBS samples were incubated with secondary antibodies in Blocking Solution (3% normal goat serum, 1% BSA, in PBS) as described above, washed with PBS and water plus Hoechst and embedded. Images were made with an Axio Imager.M2 microscope (Zeiss) using Plan-Apochromat 63×/1.4 Oil, Plan-Neofluar 40×/1.30 Oil, and Plan-Apochromat 20×/0.8 objectives and analyzed with Fiji (v 1.48g). Confocal images were acquired with a HCX PL APO lambda blue 63.0×, 1.40 NA, oil immersion objective on a Leica TCS SP5 laser scanning confocal microscope.

### Image Processing

Image deconvolution was performed using Huygens Professional Version 3.4 (Scientific Volume Imaging, Hilversum) in order to improve spatial resolution using the classical maximum likelihood estimation algorithm and a theoretical PSF. The signal to noise ratio varied between 5 and 10 in each channel.

For neuronal morphometry images were processed with Fiji (v 1.48g) and WIS-NeuroMath (v 3.4.8.; Galun et al., 2007; Rishal et al., 2013). After skeletonization of neurons the cell bodies were removed with Adobe Photoshop CS2 and the center marked with a point. Sholl analysis was carried out with the respective Fiji plug-in. The center of the cell body was defined as the center of analysis. The starting radius was set at 0 μm, the ending radius was infinite, and the radius step size was 10 μm. Neuron soma diameter was determined by measuring the shortest diameter of the cell body using the original neuron image as source. For each neuron the following morphological parameters were documented: diameter of the cell body, total neurite length (TNL), longest branch (LB), and total number of neurite intersections (TNIS).

### Data Analysis and Statistics

Statistical analysis of morphological parameters was performed using SigmaPlot 12.5 (Systat Software). A two-tailed one-sample *t*-test was applied for analysis and the results were normalized to the control and reported as mean ± SEM. A *p*-value of ≤ 0.05 was considered statistically significant.

## Results

### Nogo-B is Prominently Expressed in Schwann Cells of the Sciatic Nerve Outside the Myelin Sheath

The *nogo/reticulon4* gene is best known for its role in the large isoform Nogo-A, which is a potent myelin-associated inhibitor of regenerative nerve growth in the central nervous system (CNS). Specifically, Nogo-A has been detected at the cell surface of oligodendrocytes within the myelin sheath. It has been demonstrated to actively block axonal outgrowth by engaging a number of neuronal receptors that trigger the outgrowth inhibitory RhoA/ROCK cascade (Schweigreiter and Bandtlow, 2006; Pernet and Schwab, 2012). Nogo-B is a shorter isoform with an expression pattern and signaling characteristics distinct from Nogo-A (Oertle and Schwab, 2003; Acevedo et al., 2004). We have shown previously that Nogo-B is abundantly expressed in the PNS and that it gets proteolytically cleaved after inflicting a nerve lesion in a mouse model (Schweigreiter et al., 2007).

Following up on this finding we first aimed to identify the main cellular source of Nogo-B expression. We performed immunohistochemistry in the adult sciatic nerve and stained with neurofilament antibody for axons, MBP for the myelin sheath, and Nogo using the Bianca antiserum that recognizes both isoforms Nogo-A and -B (Figure 1A; Oertle et al., 2003b). As illustrated in Figure 1B, Nogo is prominently expressed in the external cytoplasmic compartment of Schwann cells. In contrast to Nogo-A in oligodendrocytes of the CNS (Huber et al., 2002), we did not find Nogo-B in the myelin sheath. A weak Bianca-specific signal is detected in the axons. A counter-staining of Bianca with 11C7, a monoclonal antibody that is specific for Nogo-A (Figure 1A; Oertle et al., 2003b), demonstrates that by far the largest fraction of the Schwann cell-specific Nogo immunoreactive signal is due to Nogo-B (Figure 1C). Only very low levels of 11C7 immunoreactivity were detected in Schwann cells of the adult sciatic nerve. The axonal Bianca-related immunoreactivity, on the other hand, seems to be largely due to Nogo-A, a finding that is consistent with a previous report on Nogo-A expression in peripheral axons (Huber et al., 2002). Immunocytochemistry and Western blot analysis of cultured Schwann cells verified the presence of Nogo-A, but this expression was very low relative to Nogo-B. Specifically, using the five lanes in the Western Blot of Figure 4A, we densitometrically quantified the relative expression levels of Nogo-A vs. Nogo-B with the Bianca antiserum. We found that in cultured Schwann cells the average expression level of Nogo-B is 79 times higher than that of Nogo-A (Figure 4A). To further characterize Nogo-B expression in Schwann cells, we prepared teased fibers of the sciatic nerve. As illustrated in Figure 1D, Nogo-B localizes to Cajal bands (Sherman et al., 2012b) and to a lesser extent to Schmidt-Lanterman incisures and transverse trabeculae (Court et al., 2009) confirming its distribution to the external cytoplasmic compartment in Schwann cells *in vivo*.

In sum, Nogo-B is prominently expressed in Schwann cells of the adult peripheral nerve. Other than Nogo-A in oligodendrocytes, it is not found in the myelin sheath, but is restricted to the external cytoplasmic compartment.

### Nogo-B Localizes to the Endoplasmic Reticulum and the Plasma Membrane of Schwann Cells

**Figure 2:**
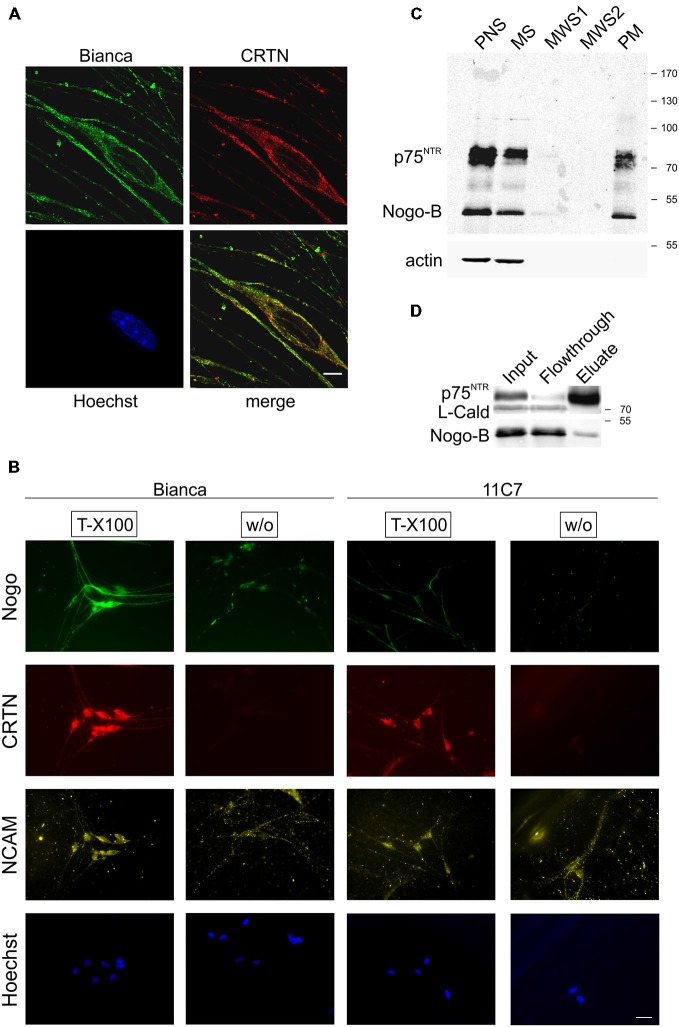
**Nogo-B localizes to the ER and the plasma membrane of Schwann cells. (A)** Confocal images of cultured primary Schwann cells. Nogo predominantly exhibits a reticular subcellular localization and co-distributes with the ER marker calreticulin (CRTN). A single confocal section is shown. Scale bar is 5 μm. **(B)** Bianca prominently stains fixed and permeabilized (Triton X-100) primary Schwann cells whereas 11C7 gives only a weak signal indicating that, similar to sciatic nerve immunohistochemistry, most of Bianca immunoreactivity is due to Nogo-B. When omitting the permeabilization step (without Triton X-100) some Bianca immunoreactivity remains indicating surface expression of Nogo-B on Schwann cells. In contrast, no 11C7 signal is seen on non-permeabilized cells demonstrating absence of Nogo-A from the Schwann cell plasma membrane. CRTN serves as a negative control for plasma membrane staining, NCAM as a positive control. Scale bar is 50 μm. **(C)** Plasma membrane sheets of primary Schwann cells were enriched and fractions of the enrichment procedure were assayed by Western blotting. Equal amounts of protein (15 μg) were loaded in postnuclear supernatant (PNS) and microsomal supernatant (MS). The membrane pellet was washed with 350 μl and a 70 μl aliquot was loaded of each of the two membrane washing supernatants (MWS1–2). The final PM pellet was resuspended in 100 μl 1× Laemmli buffer and a 25 μl aliquot was loaded. The neurotrophin receptor p75^NTR^ serves as a positive control for plasma membrane localization, actin serves as a negative control. Nogo is detected with Bianca; while Nogo-B (approx. 50 kDa) is abundant in the PNS, MS as well as PM fraction, Nogo-A (approx. 180 kDa) is not detected in either fraction. **(D)** Proteins at the surface of Schwann cells were biotinylated and fractions were analyzed by Western blotting. p75^NTR^ serves a positive control for surface expression, L-caldesmon as a negative control.

We next analyzed the subcellular distribution of Nogo-B in primary Schwann cells. First, we performed confocal microscopy; as expected for a reticulon protein we found a predominantly reticular staining pattern and a very good co-localization with the ER marker calreticulin (CRTN; Figure 2A). Since a small fraction of Nogo-A distributes to the cell surface of oligodendrocytes and neurons (Oertle et al., 2003b; Dodd et al., 2005), we investigated a potential surface localization of Nogo-B on Schwann cells. We stained permeabilized and non-permeabilized Schwann cells with Bianca and 11C7. As shown in Figure 2B, there was a very strong Nogo expression in Schwann cells as revealed by the Bianca antiserum. Similar to the sciatic nerve sections, by far the largest fraction of this Bianca immunoreactivity was due to Nogo-B because the Nogo-A-specific staining, which was performed with 11C7, was quite weak. When staining non-permeabilized Schwann cells we detected some Bianca immunoreactivity whereas the 11C7 signal was completely gone (Figure 2B). This data indicate that there is a marked fraction of Nogo-B present on the cell surface, but no Nogo-A. Because the Bianca antiserum recognizes an epitope in the amino terminus of Nogo-B (AmNogo-B) outside of the conserved reticulon homology domain (RHD; Figure 1A), we know from this cell surface staining data that the amino terminus of Nogo-B is exposed on the cell surface (Figure 2B). To confirm the surface localization of Nogo-B, we purified the plasma membrane from cultured Schwann cells and ran the fractions on a Western Blot. As illustrated in Figure 2C, we found Nogo-B in the plasma membrane fraction along with the transmembrane neurotrophin receptor p75^NTR^, but not actin, which served as a negative control. Nogo-A was not detected in either fraction presumably due to its low expression level. Finally, we performed a surface biotinylation experiment that verified the presence of Nogo-B on the surface of primary Schwann cells (Figure 2D).

In sum, this series of immunostainings and biochemical experiments demonstrates surface localization of Nogo-B in Schwann cells aside from its ER residency.

### Reduced Number of Axonal Branches of Sensory Neurons Cultured on *Nogo-a/b* Deficient Schwann Cells

**Figure 3:**
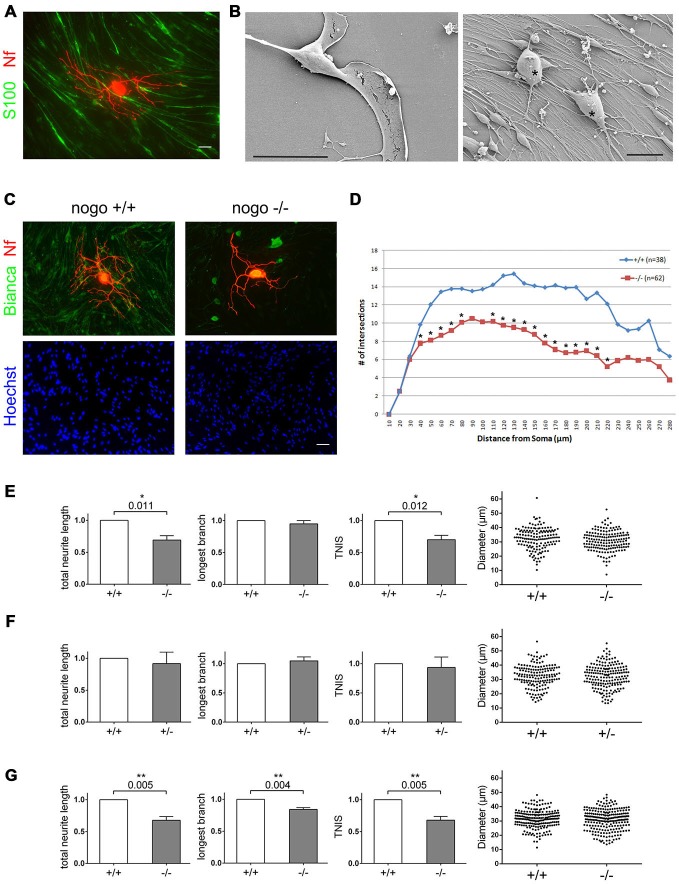
**Deletion of Nogo-B in Schwann cells reduces the extent of neuronal branch formation. (A)** DRG-derived sensory neurons are cultured on a high density Schwann cell monolayer. Schwann cells are stained with S100ß, neurons with a neurofilament antibody. Scale bar is 10 μm. **(B)** SEM images of an individual Schwann cell and the Schwann cell neuron co-culture. Neurons are marked with an asterisk. Scale bar is 20 μm. **(C)** Wildtype sensory neurons from an adult mouse are cultured on *nogo*^+/+^ and *nogo*^−/−^ Schwann cells. The co-culture is stained with Bianca, Hoechst, and a neurofilament antibody. Scale bar is 10 μm. **(D)** Sholl analysis of neurons grown on *nogo*^+/+^ vs. *nogo*^−/−^ Schwann cells. n denotes the number of neurons analyzed per Schwann cell genotype. Asterisks indicate statistically significant differences in the number of intersections per increment. **(E)** Quantification of morphological parameters of adult wildtype sensory neurons grown on *nogo*^+/+^ vs. *nogo*^−/−^ Schwann cells. TNIS denotes the total number of intersections of a neuron’s neurites in the Sholl analysis whereas TNL is the total neurite length (TNL). Five independent experiments were carried out with a total of 148 neurons being analyzed in the wildtype and 163 neurons in the knockout group. Morphological parameters were normalized to the wildtype control (set to 1.0) and expressed as mean ± SEM. *P*-values ≤ 0.05 are indicated. In the knockout TNIS is reduced to 0.71 and TNL to 0.69 relative to the wildtype levels. Neuronal cell body diameters are depicted in a dot blot. **(F)** Quantification of morphological parameters of adult wildtype sensory neurons grown on *nogo*^+/+^ vs. *nogo*^+/−^ Schwann cells. Five independent experiments were carried out with a total of 161 neurons being analyzed in the wildtype and 185 neurons in the heterozygous group. Morphological parameters were normalized to the wildtype control (set to 1.0) and expressed as mean ± SEM. *P*-values ≤ 0.05 are indicated. Neuronal cell body diameters are depicted in a dot blot. **(G)** Quantification of morphological parameters of sensory neurons obtained from wildtype p6–9 mice grown on *nogo*^+/+^ vs. *nogo*^−/−^ Schwann cells. Five independent experiments were carried out with a total of 209 neurons being analyzed in the wildtype and 232 neurons in the knockout group. Morphological parameters were normalized to the wildtype control (set to 1.0) and expressed as mean ± SEM. *P*-values ≤ 0.05 are indicated. In the knockout both TNIS and TNL are reduced to 0.68 relative to the wildtype levels. The longest branch (LB) value is reduced to 0.84 relative to wildtype. Neuronal cell body diameters are depicted in a dot blot.

Because of the substantial amounts of Nogo-B in the Schwann cell plasma membrane and because of the marked effect oligodendrocyte expressed Nogo-A has on the growth pattern of CNS neurons (Schweigreiter and Bandtlow, 2006), we wondered whether Schwann cells communicate with sensory neurons *in trans* through Nogo-B and, consequently, whether deletion of Schwann cell expressed Nogo-B would affect neurons. To test this hypothesis, we established a co-culture of Schwann cells and sensory neurons with Schwann cells forming a monolayer onto which individual sensory neurons were seeded. As illustrated in Figure 3A, Schwann cells appear as spindle-shaped cells in an immunocytochemical staining; when imaged with scanning electron microscopy (SEM), however, the ultrathin sail-like processes, which would wrap around the axon to form the myelin sheath *in vivo*, become visible (Figure 3B). In high-density Schwann cell cultures these flat processes cover the entire surface (Figure 3B).

To investigate the potential effect of Nogo-B on neuronal morphology, we cultured sensory neurons derived from the DRG of an adult wildtype mouse on Schwann cells derived from a *nogo*^+/+^ and *nogo*^−/−^ mouse. As seen in Figure 3C, neurons grown on the *nogo* deficient Schwann cells formed significantly fewer branches than neurons grown on *nogo*^+/+^ Schwann cells. We quantified this effect morphometrically and found that the number of branch points, as measured by the TNIS in a Sholl analysis, is reduced by approximately 30% on *nogo*^−/−^ Schwann cells, whereas the maximal axonal elongation was unaffected (Figure 3E). The total axonal length was reduced correspondingly as expected. The Sholl analysis demonstrated that this effect of reduced TNIS was not due to a reduced extent of axonogenesis. It was due to reduced branching of primary axons that in number did not differ between neurons grown on *nogo*^+/+^ vs. *nogo*^−/−^ Schwann cells (Figure 3D).

To rule out a possible differential survival of neuronal subpopulations on wildtype vs. knockout Schwann cells, we measured the soma diameter of each morphometrically analyzed neuron. As demonstrated in Figure 3E, we observed a normal distribution of cell body diameters without any shift between these two populations. This ruled out differential survival of neuronal subtypes. As a control, we compared neurons cultured on *nogo*^+/−^ vs. *nogo*^−/−^ Schwann cells. In this heterozygous control experiment, we did not find any significant difference in neuronal morphology between the two Schwann cell genotypes although there was a statistically insignificant trend towards a reduced extent of branching on *nogo*^+/−^ Schwann cells (Figure 3F). Although we are primarily interested in the behavior of adult neurons, we included neurons from early postnatal mice in our analysis. As depicted in Figure 3G, sensory neurons from p6–9 wildtype mice exhibited not only less branching but also reduced long-distance growth when cultured on *nogo-a/b* knockout Schwann cells. The effect on branching was in fact slightly more pronounced than with adult neurons (reduction by 32% with early postnatal neurons compared to reduction by 29% with adult neurons) indicating a stronger and at the same time less specific responsivity of early postnatal neurons.

### Effect of Nogo-B on Neuronal Morphology is Restricted to Undifferentiated Schwann Cells and is Mediated by Physical Contact between Glia and Neurons

**Figure 4:**
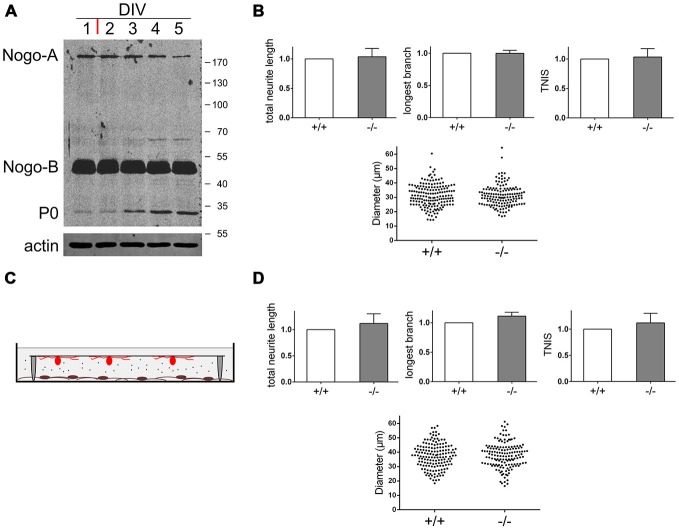
**Nogo-B’s effect on neuronal morphology depends on physical interaction with neurons and is restricted to undifferentiated Schwann cells. (A)** Western blot of a time course experiment with Schwann cells pushed towards differentiation. At DIV1 serum free medium was added plus forskolin (red bar). Equal amounts of protein (45 μg) were loaded in each lane. P0 serves as a differentiation marker, actin serves as a loading control. **(B)** Quantification of morphological parameters of adult wildtype sensory neurons grown on *nogo*^+/+^ vs. *nogo*^−/−^ Schwann cells. Sensory neurons were added at DIV4 to differentiating Schwann cells and the co-culture was fixed at DIV5. Five independent experiments were carried out with a total of 166 neurons being analyzed in the wildtype and 144 neurons in the knockout group. Morphological parameters were normalized to the wildtype control (set to 1.0) and expressed as mean ± SEM. *P*-values ≤ 0.05 are indicated. Neuronal cell body diameters are depicted in a dot blot. **(C)** Schematic of the indirect Schwann cell neuron co-culture lacking physical contact between the two cell types. Neurons were plated on a glass cover slip with paraffin feet attached and flipped face down onto the Schwann cell monolayer. **(D)** Quantification of morphological parameters of adult wildtype sensory neurons grown above *nogo*^+/+^ vs. *nogo*^−/−^ Schwann cells in an indirect co-culture. Five independent experiments were carried out with a total of 155 neurons being analyzed in the wildtype and 146 neurons in the knockout group. Morphological parameters were normalized to the wildtype control (set to 1.0) and expressed as mean ± SEM. *P*-values ≤ 0.05 are indicated. Neuronal cell body diameters are depicted in a dot blot.

The Schwann cells we used for our co-culture were derived from p3–p4 mice. At this age shortly after birth there are mostly immature Schwann cells in the promyelination stage (Sherman et al., 2012a). We wondered whether pushing Schwann cells towards differentiation, i.e., myelination, would affect the branching phenotype. We cultured Schwann cells in chemically defined medium in the absence of serum and presence of elevated cAMP for 4 days before adding adult wildtype sensory neurons. In concert with previous observations (Morgan et al., 1991), the myelination marker P0 became up-regulated during this time period (Figure 4A). The expression levels of Nogo-B and also Nogo-A, however, remained unaltered. When analyzing neurons cultured on these differentiating Schwann cells, we did not find any morphological difference between neurons grown on *nogo*^+/+^ vs. *nogo*^−/−^ Schwann cells (Figure 4B). Apparently, the effect of Nogo-B on the extent of axonal branching is restricted to immature, undifferentiated Schwann cells. This loss of phenotype cannot simply be correlated with a reduced expression level of Nogo-B (Figure 4A) or with diminished distribution of Nogo-B to the plasma membrane (data not shown). Presumably, the change in responsivity of neurons grown on differentiated vs. undifferentiated Schwann cells is due to the extensive transformation of gene expression and signaling activity that occurs during Schwann cell differentiation (Monk et al., 2015).

Next, we wanted to determine whether the Nogo-B mediated effect on axonal branching is mediated directly via physical interaction between Schwann cell expressed Nogo-B and putative neuronal receptors or indirectly via protein or exosome secretion (Corfas et al., 2004; Lopez-Verrilli and Court, 2012). The main subcellular locality of Nogo-B is the ER and therefore the secretion scenario seemed plausible. To test this hypothesis, we cultured Schwann cells as described and added adult sensory neurons on a coverslip face down. Tiny paraffin feet on the coverslip prevented physical contact between the two cell types but allowed for free chemical communication (Figure 4C). When comparing adult sensory neurons grown above *nogo*^+/+^ vs. *nogo*^−/−^ Schwann cells in this experimental set-up, we did not observe a significant difference in neuronal morphology. In particular, there was no reduced branching of axons in the absence of Nogo-B in Schwann cells (Figure 4D). However, we did notice an insignificant trend towards an increased rate of branching with *nogo-a/b* knockout Schwann cells indicating a weak influence by Nogo-B on the axonal growth pattern. This seems to be mediated in a secretion-dependent manner and to counteract the influence on branching by direct physical interaction.

In sum, using a co-culture system of wildtype and *nogo-a/b* deficient Schwann cells and adult wildtype sensory neurons shows that deletion of Nogo-B results in a marked reduction in the number of axonal branches without affecting axonal long-distance growth. This phenotype is more pronounced, but also less specific with early postnatal neurons. Furthermore, we demonstrated that this branching phenotype is mediated only by immature, undifferentiated Schwann cells in a direct manner. This implicates neuronal Nogo-B receptor(s) in signal transmission.

### Blockade of the Neuronally Expressed Nogo-B Receptor NgBR Reproduces the Loss-of-*Nogo* Branching Phenotype

**Figure 5:**
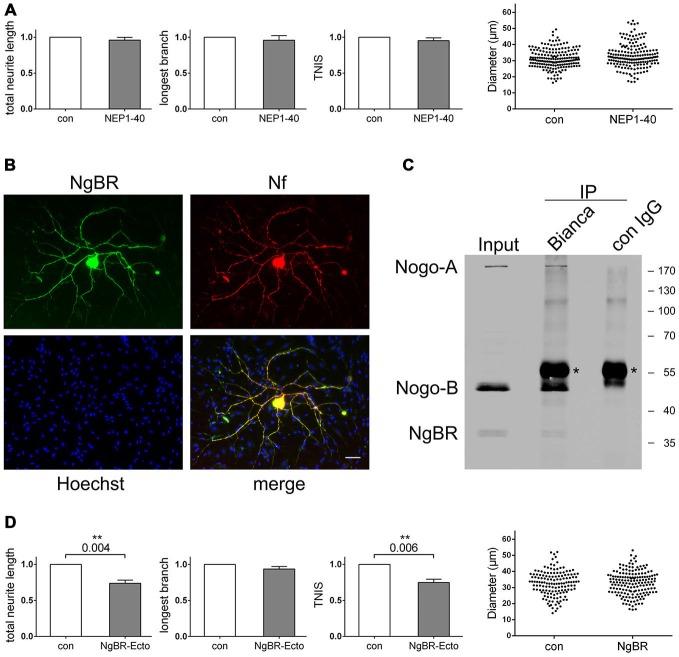
**Blocking the Nogo-B receptor NgBR mimics the loss-of-*nogo* branching phenotype. (A)** Quantification of morphological parameters of adult wildtype sensory neurons grown on wildtype Schwann cells in the presence or absence of NEP1–40. Five independent experiments were carried out with a total of 181 neurons being analyzed in the control and 177 neurons in the NEP1–40 group. Morphological parameters were normalized to the control (PBS; set to 1.0) and expressed as mean ± SEM. *P*-values ≤ 0.05 are indicated. Neuronal cell body diameters are depicted in a dot blot. **(B)** Immunocytochemistry of a Schwann cell neuron co-culture stained for NgBR, Hoechst and neurofilament. Scale bar is 10 μm. **(C)** Co-immunoprecipitation experiments with a co-culture of wildtype Schwann cells and *nogo-a/b* deficient sensory neurons. For pull-down Bianca antiserum or a rabbit control IgG was used. Detection was done with Bianca antiserum and an NgBR antibody. Asterisks indicate the rabbit IgG heavy chain that is detected by the secondary anti-rabbit antibody. **(D)** Quantification of morphological parameters of adult wildtype sensory neurons grown on wildtype Schwann cells in the presence or absence of NgBR-Ecto. Five independent experiments were carried out with a total of 142 neurons being analyzed in the control and 167 neurons in the NgBR-Ecto group. Morphological parameters were normalized to the control (50% glycerol in PBS; set to 1.0) and expressed as mean ± SEM. *P*-values ≤ 0.05 are indicated. In the presence of NgBR-Ecto TNIS is reduced to 0.75 and TNL to 0.74 relative to the control levels. Neuronal cell body diameters are depicted in a dot blot.

To identify the neuronal receptors that mediate Nogo-B’s impact on axonal branching we addressed three candidate receptors. Two of them, NgR1 and PirB, bind to the Nogo-66 loop domain within the RHD, whereas the Nogo-B receptor NgBR interacts with a site that is not yet precisely mapped but spans the RHD and the amino terminus (Figure 1A). We used the NEP1–40 peptide, which is derived from the Nogo-66 sequence and competitively blocks NgR1 (GrandPré et al., 2002) and presumably also PirB (Gou et al., 2014), to test the involvement of NgR1 and PirB. Both of these receptors are expressed in adult sensory neurons (Atwal et al., 2008; Wörter et al., 2009). If either of these receptors were to mediate the effect of Nogo-B on axonal branching, then blocking it with NEP1–40 would mimic loss of Nogo-B. We co-cultured wildtype Schwann cells and wildtype sensory neurons in the absence or presence of NEP1–40. As indicated in Figure 5A, there was no difference in any parameter of neuronal morphology; in particular, we did not observe reduced axonal branching of sensory neurons cultured in the presence of NEP1–40. Thus, we ruled out a role of NgR1 and PirB in mediating the branching effect of Nogo-B. Next, we addressed the potential role of NgBR. NgBR is less well characterized than NgR1 and PirB. It was identified as a receptor of AmNogo-B in endothelial cells and was shown to mediate Nogo-B dependent chemotaxis and morphogenesis of vascular endothelial cells (Miao et al., 2006). The relevance of NgBR to the nervous system is completely unknown as is its neuronal expression pattern, if any.

To investigate this, we tested for expression of NgBR in adult sensory neurons. As shown in Figure 5B, we found strong expression of NgBR in sensory neurons both in the soma and the axonal processes. Co-cultured Schwann cells, however, were completely negative for NgBR. We next explored the functional relationship of Schwann cell expressed Nogo-B and neuronal NgBR. We tested for a physical *in trans* interaction between these two proteins by co-immunoprecipitation (co-IP). In fact, demonstrating the interaction between endogenous Nogo-B and NgBR via co-IP has not yet been reported, but we suspected that we would have a good chance with our co-culture system given the high expression levels of these two proteins. Using wildtype Schwann cells, which expressed Nogo-B but not NgBR as well as *nogo-a/b* deficient neurons, which expressed NgBR but lacked Nogo-B, we carried out an immunoprecipitation with Bianca antiserum and detected with Bianca and for NgBR. As shown in Figure 5C, there was robust pull-down of Nogo-B plus NgBR whereas both bands were missing in a control pull-down using a non-specific rabbit IgG. We thus concluded that Schwann cell expressed Nogo-B and neuronal NgBR form an effective signaling axis. To interfere with this signaling pathway, we employed a recombinant NgBR-ectodomain (NgBR-Ecto) to outcompete and block endogenous NgBR. In a co-culture of wildtype Schwann cells and wildtype sensory neurons we observed a marked inhibitory effect of NgBR-Ecto on the extent of axonal branching without affecting long-distance growth (Figure 5D). Adding NgBR-Ecto actually mimicked both in a qualitative and quantitative manner the genetic deletion of Nogo-B in Schwann cells.

These data indicate that it is NgBR that transduces Nogo-B’s effect on axonal branching. From a clinical viewpoint this result is particularly interesting because the administration *in vivo* of a protein-based blocking agent like NgBR-Ecto to the lesion site is much easier than manipulating expression levels genetically.

## Discussion

We identified the Nogo-B/NgBR signaling axis as a major modulator of axonal branching. Blocking either Schwann cell expressed Nogo-B or its neuronal receptor NgBR resulted in a significant reduction in the number of axonal branch points of sensory neurons, but left axonal long-distance growth unaffected.

Nogo-B is a member of the ancient reticulon (RTN) family of membrane embedded proteins with homologs found throughout the eukaryotic domain (Oertle et al., 2003a). A hallmark of RTNs is their association with and the shaping of the ER via a highly conserved C-terminal sequence stretch that is termed the RHD (Voeltz et al., 2006; Hu et al., 2008). Importantly, the RHD not only is necessary but also sufficient for ER morphogenesis (Shibata et al., 2008). In contrast to the RHD, the role of the N-terminal moiety, which has been subject to a high degree of evolutionary variation, is currently much less clear. RTNs seem to be bimodal proteins; they exert a basic ER-morphogenic function through the RHD, which is shared by most if not all RTNs as well as a specific function directed by their unique N-terminus.

Nogo-B is encoded by the *nogo/rtn4* gene which, by means of alternative splicing and differential promoter usage, gives rise to three main isoforms, Nogo-A/B/C (Oertle and Schwab, 2003). Nogo-A is the largest isoform consisting of the N-terminal NiR domain, central NiG, and C-terminal RHD. Nogo-B lacks a central NiG and is structurally characterized by direct fusion of the N-terminal NiR domain to the C-terminal RHD (Figure 1A). Apart from their unique N-termini, the Nogo isoforms are specified by their expression pattern. While Nogo-A is largely confined to oligodendrocytes and neurons of the CNS (Oertle and Schwab, 2003), Nogo-B has a more widespread expression pattern but is markedly enriched in endothelial and smooth muscle cells of blood vessels (Acevedo et al., 2004) and, as we demonstrate in this study, in Schwann cells in the PNS. In contrast to Nogo-A, however, which abundantly localizes to the myelin sheath formed by oligodendrocytes (Huber et al., 2002), we have not detected any Nogo-B in Schwann cell-produced myelin.

The functional delineation of the Nogo isoforms is surprisingly complex. Nogo-A, specifically the small fraction that distributes to the plasma membrane of oligodendrocytes, is a major inhibitor of nerve growth, especially in the lesioned adult CNS (Schweigreiter and Bandtlow, 2006; Schwab and Strittmatter, 2014). Neuronal Nogo-A modulates growth cone motility and migration of CNS neurons and has been implicated in synaptic plasticity and neuronal morphogenesis (Mingorance-Le Meur et al., 2007; Montani et al., 2009; Zagrebelsky et al., 2010; Delekate et al., 2011). A prominent role of Nogo-B has been documented for the vascular system where it was demonstrated to modulate chemotactic migration of endothelial and smooth muscle cells (Acevedo et al., 2004).

We show that another major function of Nogo-B resides with Schwann cells in the PNS. Although we found that they express very large amounts of Nogo-B, we did not observe any morphological differences between wildtype and *nogo-a/b* deficient Schwann cells. Because of the high level of surface expressed Nogo-B, we hypothesized that Schwann cells communicate with sensory neurons *in trans* through Nogo-B. Schwann cells and sensory neurons form an intimate cell partnership that is characterized by intense intercellular communication (Mirsky et al., 2002; Corfas et al., 2004). Many intercellular signaling mechanisms have been identified that are mediated either by direct physical contact or in an indirect manner through secretion of molecules or release of exosomes (Lopez-Verrilli and Court, 2012). This glia-neuron crosstalk is a prime example of how two cell types control each other’s development and maintenance in a mutual manner. We considered this notion by studying the role of Nogo-B in a co-culture of Schwann cells and sensory neurons. This approach allowed us to manipulate the two cell types individually and to precisely control the experimental conditions. We thereby obtained unambiguous results that demonstrate that Schwann cell expressed Nogo-B affects neuronal morphology *in trans* and that this interaction depends on physical contact between the two cell types. Moreover, we show that only undifferentiated Schwann cells transduce this effect and that early postnatal neurons are more plastic, but also less specific in their responsivity to Schwann cells than adult ones. Finally, we provide data that indicate that the neuronal receptor that mediates Nogo-B’s signal appears to be NgBR.

In general, the Nogo receptor system is characterized by a significant level of pleiotropy which renders the system less amenable to specific manipulation. The Nogo-66 loop domain that is harbored by both Nogo-A and Nogo-B binds to two unrelated receptors, the Nogo-66 receptor NgR1 and the paired immunoglobulin receptor PirB (Schwab and Strittmatter, 2014). NgR1 is a membrane-anchored protein and requires a transmembrane co-receptor for signal transduction, which is either the neurotrophin receptor p75^NTR^ or related TAJ/TROY. NgR1 and PirB, on the other hand, not only ligate Nogo-66 but also the myelin-associated proteins MAG and OMgp. In addition, NgR1 binds the ligands APP and LGI1 (Thomas et al., 2010; Zhou et al., 2011). Neither of these receptors, however, seems to be involved in the current Nogo-B signaling paradigm because application of the Nogo-66 antagonistic peptide NEP1–40, which blocks interaction of Nogo-66 with NgR1 and most likely also with PirB, failed to reproduce the *nogo* loss-of-function branching phenotype.

The only other known receptor-binding site of Nogo-B is located at its N-terminus and called AmNogo-B. It is not well defined and comprises parts of the NiR domain and RHD (Miao et al., 2006). In fact, AmNogo-B is characterized by the fusion of the NiR domain with the RHD to create a sequence stretch that is unique for Nogo-B. AmNogo-B was shown to bind to a transmembrane protein termed Nogo-B receptor, NgBR (Miao et al., 2006). In contrast to the Nogo-66 signaling system, the AmNogo-B/NgBR signaling axis is very specific, in that no other ligand of NgBR has been reported nor has there been another receptor for AmNogo-B. In contrast to Nogo-66, manipulation of AmNogo-B signaling thus promises to be a very specific approach. By employing an antagonistic NgBR ectodomain we interfered with this signaling pathway at its top end. In contrast to compounds that target intracellular signaling effectors, this recombinant protein does not have to cross the plasma membrane nor does it likely elicit pleiotropic effects as is often the case when manipulating downstream signaling proteins. Exactly what the downstream signaling cascade of NgBR looks like is not known presently.

A developmental study in zebrafish has implicated Akt in NgBR signalling (Zhao et al., 2010), and overexpression of NgBR in vascular endothelial cells was shown to lead to an increase of Akt phosphorylation (Teng et al., 2014). This is interesting in the current context because Akt activity is known to promote branching of peripheral neurons. Specifically, the activation of the PI3K/Akt signaling module, with concurrent inhibition of glycogen synthase kinase 3ß, has been shown to increase the branching index of sensory neurons while activation of the Ras/ERK signaling cascade is supportive of axonal long-distance growth (Zhao et al., 2009; Ketschek and Gallo, 2010; Spillane et al., 2012; Klimaschewski et al., 2013; Kim et al., 2014). It is thus tempting to speculate that in neurons growing on *nogo-a/b* deficient Schwann cells, the level of activated Akt is reduced, due to missing input from NgBR ultimately resulting in reduced branching.

During the repair phase following peripheral nerve lesion, dedifferentiated Schwann cells secrete a variety of neurotrophic factors and cytokines including NGF, BDNF, NT-4/5, FGF-2, IGF-1, IL-6, and IL-1ß that support neuronal survival and regenerative growth of axons (Chen et al., 2007; Navarro et al., 2007). These factors typically bind to receptor tyrosine kinases that trigger the activation of both the PI3K/Akt and Ras/ERK signaling cascade. For this reason not only axonal elongative growth is facilitated during peripheral regeneration but also axonal sprouting (Klimaschewski et al., 2013). From a clinical viewpoint axonal long-distance growth after injury is desirable but axonal sprouting is not. A major effort to improve peripheral regeneration therefore is to disentangle these two signaling cascades at the molecular level. One approach has been to specifically promote the Ras/ERK module over the PI3K/Akt cascade. This strategy has been implemented by blocking Sprouty2, which is a negative feedback regulator of the Ras/ERK module (Marvaldi et al., 2015). Reducing Sprouty2 to heterozygous levels in sensory neurons that have been stimulated with NGF markedly improves long-distance growth without increasing axonal sprouting to the same extent. A combination of treatments with NGF and NgBR-Ecto in a heterozygous *sprouty2* mouse mutant may coax peripheral nerve fibers towards improved long-distance growth of axons with concurrent reduced branch formation. From a clinical perspective, non-genetic manipulation of effector molecules is preferred and NgBR-Ecto, as a recombinant protein that does not have to cross the plasma membrane, lends itself to an efficient *in vivo* delivery.

In conclusion, we provide evidence for a novel function of the *nogo* gene in the PNS that is implemented by the Nogo-B isoform and mediated by neuronally expressed NgBR. Genetically deleting Nogo-B from Schwann cells or blocking its receptor NgBR both results in a markedly reduced number of axonal branches of sensory neurons without affecting maximal axonal elongation. These data imply that the Nogo-B/NgBR signaling axis selectively contributes to the formation of branch points of outgrowing axons. We observed that this effect is restricted to immature, undifferentiated Schwann cells, a scenario similar to that in a nerve lesion when Schwann cells dedifferentiate and orchestrate the regenerative growth of the peripheral fibers. Our finding is of potential clinical relevance because it may represent a novel mechanism that adds to the excessive sprouting of regenerating nerve fibers. Blocking Nogo-B/NgBR on regenerating axons may reduce excessive sprouting without compromising long-distance growth and recombinant NgBR-Ecto may be a novel tool to achieve this.

## Author Contributions

CE, NJ, LW, IF, BF, SK, KP and RS performed experiments. CE, LW and RS analyzed data. LW, IF, GW, LK and RS designed experiments. BZ provided tools. RS conceived the study and wrote the manuscript.

## Funding

This work was supported by a grant from the Austrian Science Fund FWF to GW (#P23729-B11) and by a grant from the Tiroler Wissenschaftsfonds (TWF; #UNI-0404/1517) to RS, BF was supported by the FWF PhD program #W1206-B18.

## Conflict of Interest Statement

The authors declare that the research was conducted in the absence of any commercial or financial relationships that could be construed as a potential conflict of interest.

## Abbreviations

CNS: central nervous system
CRTN: calreticulin
DIV: days *in vitro*
DRG: dorsal root ganglion
ER: endoplasmic reticulum
ERK: extracellular signal regulated kinase
LB: longest branch
MAG: myelin associated glycoprotein
MBP: myelin basic protein
NgBR: Nogo-B receptor
NCAM: neural cell adhesion molecule
p: postnatal
PI3K: phosphoinositide 3 kinase
PNS: peripheral nervous system
RHD: reticulon homology domain
RTN: reticulon
TNIS: total number of neurite intersections
TNL: total neurite length.

## References

[B1] Acevedo L. Yu J. Erdjument-Bromage H. Miao R. Q. Kim J. E. Fulton D.. (2004). A new role for Nogo as a regulator of vascular remodeling. Nat. Med. 10, 382–388. 10.1038/nm1020 15034570

[B2] Allodi I. Udina E. Navarro X. (2012). Specificity of peripheral nerve regeneration: interactions at the axon level. Prog. Neurobiol. 98, 16–37. 10.1016/j.pneurobio.2012.05.005 22609046

[B3] Atwal J. K. Pinkston-Gosse J. Syken J. Stawicki S. Wu Y. Shatz C.. (2008). PirB is a functional receptor for myelin inhibitors of axonal regeneration. Science 322, 967–970. 10.1126/science.1161151 18988857

[B4] Chen Z. L. Yu W. M. Strickland S. (2007). Peripheral regeneration. Annu. Rev. Neurosci. 30, 209–233. 10.1146/annurev.neuro.30.051606.094337 17341159

[B5] Corfas G. Velardez M. O. Ko C. P. Ratner N. Peles E. (2004). Mechanisms and roles of axon-Schwann cell interactions. J. Neurosci. 24, 9250–9260. 10.1523/jneurosci.3649-04.2004 15496660 PMC6730082

[B6] Court F. A. Hewitt J. E. Davies K. Patton B. L. Uncini A. Wrabetz L.. (2009). A laminin-2, dystroglycan, utrophin axis is required for compartmentalization and elongation of myelin segments. J. Neurosci. 29, 3908–3919. 10.1523/JNEUROSCI.5672-08.2009 19321787 PMC2940832

[B7] Delekate A. Zagrebelsky M. Kramer S. Schwab M. E. Korte M. (2011). NogoA restricts synaptic plasticity in the adult hippocampus on a fast time scale. Proc. Natl. Acad. Sci. U S A 108, 2569–2574. 10.1073/pnas.1013322108 21262805 PMC3038770

[B8] de Ruiter G. C. Malessy M. J. Alaid A. O. Spinner R. J. Engelstad J. K. Sorenson E. J.. (2008). Misdirection of regenerating motor axons after nerve injury and repair in the rat sciatic nerve model. Exp. Neurol. 211, 339–350. 10.1016/j.expneurol.2007.12.023 18448099 PMC2967197

[B9] Dodd D. A. Niederoest B. Bloechlinger S. Dupuis L. Loeffler J. P. Schwab M. E. (2005). Nogo-A, -B and -C are found on the cell surface and interact together in many different cell types. J. Biol. Chem. 280, 12494–12502. 10.1074/jbc.m411827200 15640160

[B10] English A. W. (2005). Enhancing axon regeneration in peripheral nerves also increases functionally inappropriate reinnervation of targets. J. Comp. Neurol. 490, 427–441. 10.1002/cne.20678 16127712

[B11] Faroni A. Mobasseri S. A. Kingham P. J. Reid A. J. (2015). Peripheral nerve regeneration: experimental strategies and future perspectives. Adv. Drug Deliv. Rev. 82–83, 160–167. 10.1016/j.addr.2014.11.010 25446133

[B12] Galun M. Basri R. Brandt A. (2007). “Multiscale edge detection and fiber enhancement using differences of oriented means,” in Ieee 11th International Conference on Computer Vision (Rio de Janeiro: IEEE), 1–6, 722–729.

[B13] Giese G. Traub P. (1986). Induction of vimentin synthesis in mouse myeloma cells MPC-11 by 12-0-tetradecanoylphorbol-13-acetate. Eur. J. Cell Biol. 40, 266–274. 3519221

[B14] Gou Z. Mi Y. Jiang F. Deng B. Yang J. Gou X. (2014). PirB is a novel potential therapeutic target for enhancing axonal regeneration and synaptic plasticity following CNS injury in mammals. J. Drug Target. 22, 365–371. 10.3109/1061186x.2013.878939 24405091

[B15] GrandPré T. Li S. Strittmatter S. M. (2002). Nogo-66 receptor antagonist peptide promotes axonal regeneration. Nature 417, 547–551. 10.1038/417547a 12037567

[B16] Hausott B. Vallant N. Auer M. Yang L. Dai F. Brand-Saberi B.. (2009). Sprouty2 down-regulation promotes axon growth by adult sensory neurons. Mol. Cell. Neurosci. 42, 328–340. 10.1016/j.mcn.2009.08.005 19683577

[B17] Hendry I. A. Hill C. E. Watters D. J. (1986). Long-term retention of fast blue in sympathetic neurones after axotomy and regeneration-demonstration of incorrect reconnections. Brain Res. 376, 292–298. 10.1016/0006-8993(86)90192-7 3755368

[B18] Hu J. Shibata Y. Voss C. Shemesh T. Li Z. Coughlin M.. (2008). Membrane proteins of the endoplasmic reticulum induce high-curvature tubules. Science 319, 1247–1250. 10.1126/science.1153634 18309084

[B19] Huber A. B. Weinmann O. Brösamle C. Oertle T. Schwab M. E. (2002). Patterns of Nogo mRNA and protein expression in the developing and adult rat and after CNS lesions. J. Neurosci. 22, 3553–3567. 11978832 10.1523/JNEUROSCI.22-09-03553.2002 PMC6758364

[B20] Ketschek A. Gallo G. (2010). Nerve growth factor induces axonal filopodia through localized microdomains of phosphoinositide 3-kinase activity that drive the formation of cytoskeletal precursors to filopodia. J. Neurosci. 30, 12185–12197. 10.1523/JNEUROSCI.1740-10.2010 20826681 PMC2944214

[B21] Kim M. S. Shutov L. P. Gnanasekaran A. Lin Z. Rysted J. E. Ulrich J. D.. (2014). Nerve growth factor (NGF) regulates activity of nuclear factor of activated T-cells (NFAT) in neurons via the phosphatidylinositol 3-kinase (PI3K)-Akt-glycogen synthase kinase 3beta (GSK3beta) pathway. J. Biol. Chem. 289, 31349–31360. 10.1074/jbc.M114.587188 25231981 PMC4223335

[B22] Klimaschewski L. Hausott B. Angelov D. N. (2013). The pros and cons of growth factors and cytokines in peripheral axon regeneration. Int. Rev. Neurobiol. 108, 137–171. 10.1016/b978-0-12-410499-0.00006-x 24083434

[B23] Lopez-Verrilli M. A. Court F. A. (2012). Transfer of vesicles from schwann cells to axons: a novel mechanism of communication in the peripheral nervous system. Front. Physiol. 3:205. 10.3389/fphys.2012.00205 22707941 PMC3374349

[B24] Mackinnon S. E. Dellon A. L. O’Brien J. P. (1991). Changes in nerve fiber numbers distal to a nerve repair in the rat sciatic nerve model. Muscle Nerve 14, 1116–1122. 10.1002/mus.880141113 1745287

[B25] Mammen A. L. Huganir R. L. O’Brien R. J. (1997). Redistribution and stabilization of cell surface glutamate receptors during synapse formation. J. Neurosci. 17, 7351–7358. 9295381 10.1523/JNEUROSCI.17-19-07351.1997 PMC6573457

[B26] Marvaldi L. Thongrong S. Kozłowska A. Irschick R. Pritz C. O. Bäumer B.. (2015). Enhanced axon outgrowth and improved long-distance axon regeneration in sprouty2 deficient mice. Dev. Neurobiol. 75, 217–231. 10.1002/dneu.22224 25104556

[B27] Miao R. Q. Gao Y. Harrison K. D. Prendergast J. Acevedo L. M. Yu J.. (2006). Identification of a receptor necessary for Nogo-B stimulated chemotaxis and morphogenesis of endothelial cells. Proc. Natl. Acad. Sci. U S A 103, 10997–11002. 10.1073/pnas.0602427103 16835300 PMC1544163

[B28] Mingorance-Le Meur A. Zheng B. Soriano E. del Río J. A. (2007). Involvement of the myelin-associated inhibitor Nogo-A in early cortical development and neuronal maturation. Cereb. Cortex 17, 2375–2386. 10.1093/cercor/bhl146 17192421

[B29] Mirsky R. Jessen K. R. Brennan A. Parkinson D. Dong Z. Meier C.. (2002). Schwann cells as regulators of nerve development. J. Physiol. Paris 96, 17–24. 10.1016/s0928-4257(01)00076-6 11755779

[B30] Monk K. R. Feltri M. L. Taveggia C. (2015). New insights on schwann cell development. Glia 63, 1376–1693. 10.1002/glia.22852 25921593 PMC4470834

[B31] Montani L. Gerrits B. Gehrig P. Kempf A. Dimou L. Wollscheid B.. (2009). Neuronal Nogo-A modulates growth cone motility via Rho-GTP/LIMK1/cofilin in the unlesioned adult nervous system. J. Biol. Chem. 284, 10793–10807. 10.1074/jbc.M808297200 19208621 PMC2667767

[B32] Morgan L. Jessen K. R. Mirsky R. (1991). The effects of cAMP on differentiation of cultured Schwann cells: progression from an early phenotype (04+) to a myelin phenotype (P0+, GFAP-, N-CAM-, NGF-receptor-) depends on growth inhibition. J. Cell Biol. 112, 457–467. 10.1083/jcb.112.3.457 1704008 PMC2288828

[B33] Navarro X. Vivó M. Valero-Cabré A. (2007). Neural plasticity after peripheral nerve injury and regeneration. Prog. Neurobiol. 82, 163–201. 10.1016/j.pneurobio.2007.06.005 17643733

[B34] Oertle T. Klinger M. Stuermer C. A. Schwab M. E. (2003a). A reticular rhapsody: phylogenic evolution and nomenclature of the RTN/Nogo gene family. FASEB J. 17, 1238–1247. 10.1096/fj.02-1166hyp 12832288

[B36] Oertle T. van der Haar M. E. Bandtlow C. E. Robeva A. Burfeind P. Buss A.. (2003b). Nogo-A inhibits neurite outgrowth and cell spreading with three discrete regions. J. Neurosci. 23, 5393–5406. 12843238 10.1523/JNEUROSCI.23-13-05393.2003 PMC6741224

[B35] Oertle T. Schwab M. E. (2003). Nogo and its paRTNers. Trends Cell Biol. 13, 187–194. 10.1016/s0962-8924(03)00035-7 12667756

[B37] Olpe H. R. Karlsson G. Pozza M. F. Brugger F. Steinmann M. Van Riezen H.. (1990). CGP 35348, a centrally active blocker of GABAB receptors. Eur. J. Pharmacol. 187, 27–38. 10.1016/0014-2999(90)90337-6 2176979

[B38] Pernet V. Schwab M. E. (2012). The role of Nogo-A in axonal plasticity, regrowth and repair. Cell Tissue Res. 349, 97–104. 10.1007/s00441-012-1432-6 22588543

[B39] Rishal I. Golani O. Rajman M. Costa B. Ben-Yaakov K. Schoenmann Z.. (2013). WIS-NeuroMath enables versatile high throughput analyses of neuronal processes. Dev. Neurobiol. 73, 247–256. 10.1002/dneu.22061 23055261

[B40] Schwab M. E. Strittmatter S. M. (2014). Nogo limits neural plasticity and recovery from injury. Curr. Opin. Neurobiol. 27, 53–60. 10.1016/j.conb.2014.02.011 24632308 PMC4122629

[B41] Schweigreiter R. Bandtlow C. E. (2006). Nogo in the injured spinal cord. J. Neurotrauma 23, 384–396. 10.1089/neu.2006.23.384 16629624

[B42] Schweigreiter R. Stasyk T. Contarini I. Frauscher S. Oertle T. Klimaschewski L.. (2007). Phosphorylation-regulated cleavage of the reticulon protein Nogo-B by caspase-7 at a noncanonical recognition site. Proteomics 7, 4457–4467. 10.1002/pmic.200700499 18072206

[B43] Schweigreiter R. Walmsley A. R. Niederöst B. Zimmermann D. R. Oertle T. Casademunt E.. (2004). Versican V2 and the central inhibitory domain of Nogo-A inhibit neurite growth via p75NTR/NgR-independent pathways that converge at RhoA. Mol. Cell. Neurosci. 27, 163–174. 10.1016/j.mcn.2004.06.004 15485772

[B44] Sherman D. L. Krols M. Wu L. M. Grove M. Nave K. A. Gangloff Y. G.. (2012a). Arrest of myelination and reduced axon growth when Schwann cells lack mTOR. J. Neurosci. 32, 1817–1825. 10.1523/JNEUROSCI.4814-11.2012 22302821 PMC4298696

[B45] Sherman D. L. Wu L. M. Grove M. Gillespie C. S. Brophy P. J. (2012b). Drp2 and periaxin form Cajal bands with dystroglycan but have distinct roles in Schwann cell growth. J. Neurosci. 32, 9419–9428. 10.1523/JNEUROSCI.1220-12.2012 22764250 PMC3400949

[B46] Shibata Y. Voss C. Rist J. M. Hu J. Rapoport T. A. Prinz W. A.. (2008). The reticulon and DP1/Yop1p proteins form immobile oligomers in the tubular endoplasmic reticulum. J. Biol. Chem. 283, 18892–18904. 10.1074/jbc.M800986200 18442980 PMC2441541

[B47] Spillane M. Ketschek A. Donnelly C. J. Pacheco A. Twiss J. L. Gallo G. (2012). Nerve growth factor-induced formation of axonal filopodia and collateral branches involves the intra-axonal synthesis of regulators of the actin-nucleating Arp2/3 complex. J. Neurosci. 32, 17671–17689. 10.1523/JNEUROSCI.1079-12.2012 23223289 PMC3596264

[B48] Sumner A. J. (1990). Aberrant reinnervation. Muscle Nerve 13, 801–803. 10.1002/mus.880130905 2233866

[B49] Teng R. J. Rana U. Afolayan A. J. Zhao B. Miao Q. R. Konduri G. G. (2014). Nogo-B receptor modulates angiogenesis response of pulmonary artery endothelial cells through eNOS coupling. Am. J. Respir. Cell Mol. Biol. 51, 169–177. 10.1165/rcmb.2013-0298OC 24568601 PMC4148038

[B50] Thomas R. Favell K. Morante-Redolat J. Pool M. Kent C. Wright M.. (2010). LGI1 is a Nogo receptor 1 ligand that antagonizes myelin-based growth inhibition. J. Neurosci. 30, 6607–6612. 10.1523/JNEUROSCI.5147-09.2010 20463223 PMC6632578

[B51] Voeltz G. K. Prinz W. A. Shibata Y. Rist J. M. Rapoport T. A. (2006). A class of membrane proteins shaping the tubular endoplasmic reticulum. Cell 124, 573–586. 10.1016/j.cell.2005.11.047 16469703

[B52] Walko G. Wögenstein K. L. Winter L. Fischer I. Feltri M. L. Wiche G. (2013). Stabilization of the dystroglycan complex in Cajal bands of myelinating Schwann cells through plectin-mediated anchorage to vimentin filaments. Glia 61, 1274–1287. 10.1002/glia.22514 23836526

[B53] Wörter V. Schweigreiter R. Kinzel B. Mueller M. Barske C. Böck G.. (2009). Inhibitory activity of myelin-associated glycoprotein on sensory neurons is largely independent of NgR1 and NgR2 and resides within Ig-Like domains 4 and 5. PLoS One 4:e5218. 10.1371/journal.pone.0005218 19367338 PMC2666269

[B54] Wrabetz L. Feltri M. L. Kim H. Daston M. Kamholz J. Scherer S. S.. (1995). Regulation of neurofibromin expression in rat sciatic nerve and cultured Schwann cells. Glia 15, 22–32. 10.1002/glia.440150104 8847098

[B55] Zagrebelsky M. Schweigreiter R. Bandtlow C. E. Schwab M. E. Korte M. (2010). Nogo-A stabilizes the architecture of hippocampal neurons. J. Neurosci. 30, 13220–13234. 10.1523/JNEUROSCI.1044-10.2010 20926648 PMC6634728

[B56] Zhao B. Chun C. Liu Z. Horswill M. A. Pramanik K. Wilkinson G. A.. (2010). Nogo-B receptor is essential for angiogenesis in zebrafish via Akt pathway. Blood 116, 5423–5433. 10.1182/blood-2010-02-271577 20813898 PMC3012551

[B57] Zhao Z. Wang Z. Gu Y. Feil R. Hofmann F. Ma L. (2009). Regulate axon branching by the cyclic GMP pathway via inhibition of glycogen synthase kinase 3 in dorsal root ganglion sensory neurons. J. Neurosci. 29, 1350–1360. 10.1523/JNEUROSCI.3770-08.2009 19193882 PMC2868143

[B58] Zheng B. Ho C. Li S. Keirstead H. Steward O. Tessier-Lavigne M. (2003). Lack of enhanced spinal regeneration in Nogo-deficient mice. Neuron 38, 213–224. 10.1016/s0896-6273(03)00225-3 12718856

[B59] Zhou X. Hu X. He W. Tang X. Shi Q. Zhang Z.. (2011). Interaction between amyloid precursor protein and Nogo receptors regulates amyloid deposition. FASEB J. 25, 3146–3156. 10.1096/fj.11-184325 21670066 PMC3157691

